# Persistent Coagulopathy After Synthetic Cannabinoid Use

**DOI:** 10.7759/cureus.36156

**Published:** 2023-03-14

**Authors:** Mahvish Haider, Carlos Acevedo-Cajigas, Desiree Ortiz, Christian A Zorrilla, Jorge Perez

**Affiliations:** 1 Internal Medicine, HCA Florida Brandon Hospital, Brandon, USA

**Keywords:** vitamin k1, brodafacoum, superwarfarin, long-acting anticoagulant, synthetic cannabinoids

## Abstract

Synthetic cannabinoids (SCs) are chemical compounds created and manufactured, without quality control standards or requirements, to mimic tetrahydrocannabinol (THC). They are widely available in the USA, and they are sold under various brand names, including “K2” and “spice.” Many adverse effects have been attributed to SCs, but most recently, they have also been associated with bleeding. There have been reported cases around the globe of SCs contaminated with long-acting anticoagulant rodenticide (LAAR) or superwarfarins. They are developed from compounds such as bromethalin, brodifacoum (BDF), and dicoumarol. LAAR exhibits their mechanism as a vitamin K antagonist inhibiting vitamin K 2,3-epoxide reductase, preventing activation of vitamin K1 (phytonadione). Therefore, reducing the activation of clotting factors II, VII, IX, and X and proteins C and S. In contrast to warfarin, BDF has an extremely long-acting biological half-life of 90 days due to minimal metabolism and limited clearance. Here, we report a 45-year-old male who presented to the emergency room with a 12-day history of gross hematuria and mucosal bleeding without previous history of coagulopathy and recurrent SCs use.

## Introduction

Synthetic cannabinoids (SCs) are chemical compounds created and manufactured, without quality control standards or requirements, to mimic tetrahydrocannabinol (THC). They are widely available in the USA, and they are sold under various brand names, including “K2” and “spice.” These products are often mixed with plant material to be smoked, therefore providing a route of administration. The federal government has banned many specific SCs. Manufacturers often change their chemical formulas labeling under “not for human consumption,” in order to avoid criminal prosecution [1].

Many adverse effects have been attributed to SCs intoxication, such as hallucinations, agitation, anxiety, tachycardia, and elevated blood pressure. Still, most recently, they have also been associated with bleeding due to contamination of SCs with long-acting anticoagulant rodenticide (LAAR) or superwarfarins [2]. These are lipophilic, vitamin k antagonists derived from warfarin or 1,3 indandione that are up to 100 times as potent as clinically used warfarin [3,4]. They are developed from compounds such as bromethalin, brodifacoum (BDF), and dicoumarol. LAAR toxication can cause prolonged coagulopathy due to their extensive half lifes or life-threatening hemorrhage if large amounts are ingested [5]. Here, we report a 45-year-old male who presented to the emergency room with a 12-day history of gross hematuria and mucosal bleeding without previous history of coagulopathy and recurrent SCs use.

## Case presentation

A 45-year-old male with no significant past medical history presented to the emergency room with a 12-day history of recurrent painless hematuria and gingival bleeding. He had been admitted to another hospital with similar symptoms and was treated with vitamin K supplementation for four days due to abnormal supratherapeutic international normalized ratio (INR) levels. However, the patient was discharged against medical advice from that hospital. He decided to come to our medical facility to be re-evaluated due to the recurrence of symptoms. The patient denied any family history of bleeding or thrombotic disorders. He reports a one-year history of weekly use of SCs. The last use was 14 days earlier. Upon physical examination, the patient experienced mild tenderness at the right costovertebral angle and mild gingival bleeding was present.

Initial laboratory tests showed hemoglobin=15.1 g/dL, platelet count= 242 x 10^3^ µL, and White blood cells count= 8.8 x 10^3^ µL. Urinalysis was positive for blood, and microscopic examination revealed >100 red blood cells per high power field. Liver and kidney function tests were within normal limits. The Hepatitis panel was negative. The urine toxicology screen was only positive for THC. Coagulation studies were performed: activated partial thromboplastin time (aPTT) of 61 seconds, international normalized ratio (INR), and prothrombin time (PT) results were not determinable.

LAAR intoxication was suspected due to recent SCs use by the patient and similar cases being admitted to the hospital with unknown etiology of bleeding and “indeterminable” INR. The local poison control department was contacted, and based on their advice, the patient was administered 10 mg of IV vitamin K1 (phytonadione). Blood samples were subsequently sent for the qualitative detection of superwarfarin components with high-performance liquid chromatography/tandem mass spectrometry (LC-MS/MS). The decision to hold 4-factor prothrombin complex concentrate treatment was based upon a lack of laboratory values for INR and non-life-threatening bleeding. A repeated INR and PT four hours after administration of intravenous phytonadione showed values of 3.4 and 40 seconds, respectively. Based on the clinical history and patient’s response to treatment, the vitamin K1 dose was increased to 150 mg daily. INR/PT normalized after eight days of constant administration of vitamin K1. There was no evidence of active bleeding during the rest of the hospital course. Due to the elevated cost of vitamin K1 supplementation, a dose reduction strategy provided by the local poison control department was initiated. Our goal was to decrease the dosage of vitamin K1 to 50 mg or less and reduce the INR to 1.3 before providing a safe discharge to the patient. However, the patient decided to leave against medical advice 48 hours after the initiation of protocol. He was prescribed vitamin k 1 (100mg daily). Attempts were initiated with the local Health Department office to provide a low-cost vitamin K supplementation. Eventually, superwarfarin panel testing using LC-MS/MS technique was negative for Warfarin and positive for BDF.

## Discussion

BDF is a potent, long-acting anticoagulant rodenticide (LAAR) or “superwarfarin” that can cause fatal poisoning due to life-threatening bleeding in humans [6]. It acts as a vitamin K antagonist inhibiting vitamin K 2,3-epoxide reductase, preventing activation of vitamin K1. Therefore, reducing the activation of clotting factors II, VII, IX, and X and proteins C and S. In contrast to warfarin, BDF has an exceptionally long-acting biological half-life of 90 days due to minimal metabolism and limited clearance [7]. Although no longer available for residential consumers, products containing BDF are still being used for commercial pest control and structural pest control markets [8]. Coagulopathy due to intoxication with the use of LAAR is still an uncommon diagnosis and challenging to identify and obtain in the clinical setting, although there is growing popularity in the use of SCs recently [9].

BDF can be absorbed via inhalation, ingestion, or direct skin contact. Previous reports have linked the use of SCs and LAAR, most recently a 2018 outbreak in Illinois with 174 confirmed cases, including five deaths [10]. Despite that there is no clear evidence yet, lacing SCs with LAAR may reduce the drug's metabolism, thus prolonging the euphoria effects [11]. A thorough medical history needs to be obtained to identify possible contamination sources and differentiate between intended versus incidental exposure. The most common bleeding symptoms include hematuria, gingival bleeding, epistaxis, and gastrointestinal bleeding. A confirmatory test can be obtained using LC-MS/MS, a qualitative analysis of warfarin and superwarfarin-type agents [12].

Currently, there is no standard long-term treatment for LAAR intoxication. However, recent literature findings suggest a range of oral vitamin K1 of 50-200 mg/day with a minimum of 109 total days of duration [13]. Nevertheless, providing long-term treatment remains challenging due to high treatment costs and frequent monitoring. Our local poison control agency provided a dose reduction strategy, and a dosage of 100 mg vitamin K1 daily was achieved without any recurrence of bleeding. The estimated cost of this patient's treatment was $1,500 to $3,000 per day, without considering additional cost factors such as frequent laboratory testing, availability of the product, and outpatient follow-up with a specialist.

## Conclusions

In this case, we emphasize that with recent outbreaks emerging, LAAR intoxication should be kept high on the differential list when a patient presents with unexplained painless bleeding and vitamin K-dependent coagulopathy. In addition, patients under long-term treatment with vitamin K1 should be educated on the importance of medical compliance to prevent the recurrence of symptoms. Raising public awareness and recognition of this condition could be an essential step to developing new treatment strategies to reduce long-term duration and cost.
